# Supplementary material for: The Ca2+-activated K+ current of human sperm is mediated by Slo3
Source: eLife. 2014 Mar 26;3:e01438. doi: 10.7554/eLife.01438 (PMC3966514; doi:10.7554/eLife.01438)
Supplement: Supplementary file 1. — Indicators of merit for the mass spectrometric results. DOI: http://dx.doi.org/10.7554/eLife.01438.020 [file elife01438s001.pdf]

Supplementary File 1: Indicators of merit for the mass spectrometric results

Protein (SwissProt-ID)	Sequence coverage [%]	Peptide sequence	Charge state	Mass (m/z [Da])	ΔMass [ppm]	Modification	XCorr[1]	ΔScore[2]	Ions matched
**CatSper2** (Q96P56)	1.70	IEQISHAQR	2	541.29077	-0.61		2.95	1.00	14/16
**CatSperβ** (Q9H7T0)	16.04	FPITQYPVSLEIINEDGR	2	1046.03870	-2.71		3.97	1.00	20/34
		LTLDSQVVQALFEDTDIEK	2	1082.55322	-1.34		3.91	1.00	16/36
		FPSSFSSPVGMVFHPR	3	593.62695	-1.16		3.75	1.00	26/60
		IFPTVLTFLVDQER	2	839.46411	-0.10		3.22	1.00	22/26
		YSAVGSVTER	2	534.76996	0.55		2.80	1.00	13/18
		EPILQWTPGDVIPESEISK	2	1069.55249	-1.86		2.77	1.00	14/36
		FKFPITQYPVSLEIINEDGR	3	789.41681	-1.39		2.73	1.00	23/76
		FPSSFSSPVGmVFHPR	3	598.95996	1.14	M11 (Ox)	2.56	1.00	23/60
		DSQGFNLIK	2	511.26999	1.34		2.36	1.00	12/16
		TVVLPGYSSFLITSILDNK	2	1034.07312	0.80		2.25	1.00	13/36
		ILWLVDIPR	2	562.84479	-0.36		1.59	1.00	10/16
		FEASGPPTAFGNSR	2	719.33936	-3.03		2.04	1.00	17/26
		HTVPENIKR	3	365.20944	2.25		1.98	1.00	15/32
		YIDLGNSYVLK	2	642.84521	-0.24		1.78	1.00	10/20
		TAIASVSTLR	2	509.79694	-2.54		1.62	1.00	12/18
**CatSperδ** (Q86XM0)	3.01	VFNLIQDVQGDR	2	702.36731	-0.34		2.49	1.00	13/22
		WPDVQYQILGGR	2	716.36945	-4.45		2.14	1.00	12/22
**CatSperγ** (Q6ZRH7)	4.83	IYNLESAYELPER	2	798.89923	0.45		3.05	1.00	16/24
		DYSEDEIYR	2	595.25555	2.55		2.21	1.00	11/16
		IEQLQIQmEAAPFR	2	845.43573	0.73	M8 (Ox)	2.00	1.00	17/26
		ENFIYLADFPK	2	678.84485	-0.78		2.30	1.00	15/20
		TNSLIWTTR	2	546.29498	-1.59		2.07	1.00	12/16
**Hv1** (Q96D96)	8.79	AAAPDVAPAPGPAPR	3	453.24515	-1.09		2.70	1.00	28/56
		QmNVQLAAK	2	509.77200	1.65	M2 (Ox)	2.17	1.00	10/16
**IZUMO1** (Q8IYV9)	31.14	DFQELSLNEDAYmGVVDEATLQK	2	1316.11157	1.02	M13 (Ox)	5.03	1.00	22/44
		EEKPSPNIVTPGEATTESSISLQPLQPEK	3	1036.19873	0.79		4.43	1.00	28/112
		SLEKDYLPGHLDAK	2	793.41461	-0.10		3.86	1.00	21/26
		SSLFGLGSGAAEQTQVPK	2	888.96100	1.11		3.51	1.00	20/34
		EATLTKPmVGPEDAGSYR	2	969.46741	0.06	M8 (Ox)	2.77	1.00	15/34
		KVIDFIK	2	431.77380	0.72		2.37	1.00	11/12
**IZUMO2** (Q6UXV1)	26.70	VETNQLDLVASFVK	2	781.92371	-1.53		4.19	1.00	21/26
		AGAVLMGMEGPFFR	2	741.86432	-1.32		3.41	1.00	18/26
		FQLEQLQAR	2	566.80829	-1.48		3.04	1.00	15/16
		DEPLLEELVTLR	2	713.89294	-0.17		2.76	1.00	18/22
		AGAVLmGmEGPFFR	2	757.85889	-1.75	M6,M8 (Ox)	2.66	1.00	15/26
		VALQYQMDSK	2	591.79395	-1.49		2.49	1.00	15/18
**IZUMO3** (Q5VZ72)	15.48	FIEDVGSLLGNLIPSEVPGR	2	1056.56885	-1.54		2.73	1.00	13/38
		KLEELMGK	2	474.26440	-1.65		2.38	1.00	13/14

		VLAVQQVVK	2	492.31616	0.21		2.38	1.00	12/16
**IZUMO4** (Q1ZYL8)	45.69	EKLDQVATAVYQMMDQLYQGK	2	1230.09912	-1.48		5.71	1.00	24/40
		SWWVGDIPVSGALLTDWSDDTMK	2	1290.10681	-2.35		5.14	1.00	26/44
		EKLDQVATAVYQmmDQLYQGK	3	831.06519	-1.38	M13,M14 (Ox)	4.70	1.00	32/80
		EKLDQVATAVYQMmDQLYQGK	3	825.73328	-1.72	M14 (Ox)	4.68	0.02	31/80
		EKLDQVATAVYQmMDQLYQGK	3	825.73383	-1.05	M13 (Ox)	4.51	0.08	31/80
		SWWVGDIPVSGALLTDWSDDTmK	2	1298.10559	-1.31	M22 (Ox)	4.39	1.00	22/44
		EQVHLIQNAIIESR	2	825.45215	0.01		4.25	1.00	21/26
		SAVQGLLNYINNWHK	2	878.95862	-1.72		4.22	1.00	17/28
		LDQVATAVYQmmDQLYQGK	3	745.35315	-0.88	M11, M12 (Ox)	4.14	1.00	30/72
		MYFPGYFPNELR	2	767.36157	-2.40		2.97	1.00	13/22
		ATPAFLVSPALR	2	621.86285	-1.71		2.67	1.00	15/22
		mYFPGYFPNELR	2	775.35931	-2.01	M1 (Ox)	2.65	1.00	15/22
		ELHLAIPAK	2	496.30063	0.44		2.43	1.00	13/16
**Na^{+}/K^{+}-ATPase α4** (Q13733)	27.60	LGAVVAVTGDGVNDSPALK	2	891.98413	0.71		5.69	1.00	25/36
		QLDQILQNHPEIVFAR	2	961.01837	0.19		4.86	1.00	18/30
		GVGIISEGTETAEEVAAR	2	894.95258	0.21		4.42	1.00	21/34
		EAFQNAYLELGGLGER	2	883.93854	-0.61		4.36	1.00	22/30
		FIEQSYSSVAEmR	2	781.86133	-0.14	M12 (Ox)	4.26	1.00	20/24
		SPDFTHENPLETR	2	771.86255	-0.36		4.14	1.00	17/24
		VIMVTGDHPITAK	2	691.37964	0.82		3.80	1.00	20/24
		VDNSSLTGESEPQSR	2	803.37109	-0.15		3.71	1.00	21/28
		FIEQSYSSVAEMR	2	773.86377	-0.27		3.63	1.00	20/24
		EDSSQTHVLMMK	2	703.32562	-0.79		3.59	1.00	15/22
		GIVIATGDSTVmGR	2	696.86121	-0.07	M12 (Ox)	3.36	1.00	19/26
		GIVIATGDSTVMGR	2	688.86420	0.59		3.30	1.00	20/26
		EDSSQTHVLmmK	2	719.32007	-1.43	M10, M11 (Ox)	3.30	1.00	16/22
		RATTGDASESALLK	2	710.37555	-0.03		3.30	1.00	16/26
		KGTVAPHDQSPR	2	646.83917	0.34		3.30	1.00	14/22
		SSDTWFMLAR	2	607.28754	0.59		3.19	1.00	16/18
		EVVmDDHKLTLEELSTK	3	668.33789	-0.56	M4 (Ox)	3.18	1.00	24/64
		NmVPQQALVIR	2	642.85858	0.43	M2 (Ox)	3.06	1.00	16/20
		ATTGDASESALLK	2	632.32501	0.00		3.01	1.00	18/24
		SSDTWFmLAR	2	615.28516	0.85	M7 (Ox)	3.01	1.00	17/18
		AIVVHGAELKDIQSK	3	536.64172	-0.62		2.95	1.00	19/56
		VImVTGDHPITAK	2	699.37622	-0.44	M3 (Ox)	2.82	1.00	15/24
		ANQEILPIAK	2	548.82092	-1.43		2.69	1.00	14/18
		AIVVHGAELK	2	518.81085	-0.56		2.68	1.00	14/18
		VAEIPFNSTNK	2	610.31958	0.08		2.58	1.00	13/20
		ADIGIAmGISGSDVSK	2	768.88086	-1.99	M7 (Ox)	2.50	1.00	16/30
		NSLFQQGmR	2	548.76440	0.98	M8 (Ox)	2.42	1.00	12/16
		NSLFQQGMR	2	540.76636	-0.10		2.35	1.00	11/16
**PMCA4**	18.61	LKTSPVEGLSGNPADLEK	3	618.99774	-1.16		5.34	1.00	34/68

(P23634)		KADVGFAmGIAGTDVAK	2	833.92670	-0.53	M8 (Ox)	5.32	1.00	26/32
		EASDIILTDDNFTSIVK	2	940.97821	0.16		4.97	1.00	22/32
		QVVAVTGDGTNDGPALK	2	821.42560	-0.25		4.26	1.00	22/32
		SLDKDPmLLSGTHVmEGSGR	3	721.34485	-0.81	M7, M15 (Ox)	4.22	1.00	32/76
		TSPVEGLSGNPADLEK	2	807.40381	-0.90		4.02	1.00	22/30
		IDESSLTGESDHVK	2	758.86005	0.13		3.86	1.00	18/26
		TPLLDEEEEENPDKASK	3	648.64069	-0.32		3.83	1.00	23/64
		ADVGFAmGIAGTDVAK	2	769.87897	-0.89	M7 (Ox)	3.71	1.00	21/30
		NEKGEVEQEKLDK	2	773.39178	0.47		3.62	1.00	18/24
		IDESSLTGESDHVKK	2	822.90704	-0.47		3.59	1.00	17/28
		YGDLLPADGILIQGNDLK	3	639.01019	-0.62		3.55	1.00	25/68
		EAGHGTTKEEITK	2	700.85413	-0.49		3.40	1.00	14/24
		QVVAVTGDGTNDGPALKK	3	590.65106	-0.38		3.15	1.00	23/68
		DAEGLDEIDHAEmELR	3	620.27441	-2.03	M13 (Ox)	3.11	1.00	26/60
		GIIDSTVGEHR	2	592.30682	-0.22		3.00	1.00	15/20
		MVTGDNINTAR	2	596.29303	0.06		2.97	1.00	19/20
		KGEAVPFK	2	438.25278	-0.35		2.89	1.00	13/14
		GEVEQEKLDK	2	587.80048	-1.19		2.84	1.00	15/18
		SSPTDKHTLVK	2	606.83276	-0.06		2.50	1.00	12/20
		NKDRDDMVR	2	574.77734	-0.30		2.44	1.00	12/16
**Slo3**	5.48	LLELPQILQILR	2	724.96442	1.09		2.54	1.00	18/22
(A8MYU2)		IIDEEELNPENK	2	721.85370	-0.61		3.36	1.00	17/22
		LGLLSLHETILSDVNPR	3	626.35583	2.01		3.84	1.00	28/64
		IIIQILQSHNK	2	653.89545	-0.44		3.17	1.00	17/20
		FVITRPANEFK	2	661.36639	-0.64		2.54	1.00	14/20
**LRRC52**	10.54	QLTEYPLDIPLNTR	2	836.94952	0.75		2.93	1.00	17/26
(Q8N7C0)		SSEEDEDEAGTR	2	662.76019	-0.36		2.66	1.00	15/22
		LFLNENR	2	453.24359	-4.50		2.13	1.00	10/12

[1] XCorr: scores the number of fragment ions that are common to two different peptides with the same precursor mass and calculates the cross-correlation score for all candidate peptides queried from the database.

[2] ΔScore: measure of the difference between the top two scores for the peptides identified by that spectrum. ΔScore = (Score(Rank n Peptide) – Score(Rank 1 Peptide))/Score(Rank 1 Peptide). If no peptide is ranked 2, the peptide with rank 1 displays a delta score of 1.0. The same is true when the peptides with ranks 1 to n have the same score and the same sequence, but there is no peptide with rank n + 1. In this case, all peptides with rank 1 to n display a delta score of 1.0.
